# Physical Characteristics of Fast Roping in British Elite Law Enforcement Officers

**DOI:** 10.1002/ejsc.70134

**Published:** 2026-02-05

**Authors:** Joseph Warwick, Sarita Harris, Hannah Ranger, Paul Read, Flaminia Ronca

**Affiliations:** ^1^ University College London London UK; ^2^ St Mary’s University, Twickenham London UK

**Keywords:** exercise, injury & prevention, musculoskeletal, physiology, strength

## Abstract

Elite law enforcement and special forces operators around the world have a unique skill set, including some risky methods of entry into a scene of operation. With fast roping being actively utilized by this population, it is important to gain an understanding of the physical demands of the task. Thirty‐seven Law Enforcement Officers (LEOs) completed five 20 ft fast rope descents onto a force platform, three in standard uniform (without kit) and two with the additional weight of operational kit (with kit). Additionally, 12 LEOs were also fitted with electromyography on their dominant arm. Landing forces with and without kit showed no significant difference. Participants with hang test time (HTT) < 35s showed significantly poorer Landing Control (LC) when descending with kit (*p* < 0.01). Landing control played an important role, with peak landing force significantly higher (*p* = 0.025) in uncontrolled landings. CMJ breaking impulse and rebound jump height are the main physical measurable predictors. However, when adjusting for body weight and kit, only rebound jump height remained predictive with marginal significance (*p* = 0.06) (*R* ^*2*^ = 0.45, *p* = 0.008). The biceps brachii (BB) exhibited greater activation when descending with kit (*p* = 0.003). However, the extensor carpi radialis exhibited the greatest activation during descents in both conditions (*p* < 0.003). Landing impact forces were not significantly different between groups, however longer HTT correlated with more controlled descents and reduced landing forces. The ECR was observed to be the muscle with the highest activation on all descents, with only the BB increasing in kit.

## Highlights

- Controlled fast rope descents showed no significant difference in landing forces with or without operational kit, indicating safe execution is achievable regardless of load.
- Law enforcement Officers with hang test time (HTT) > 35 s demonstrated significantly better landing control, especially when descending with kit.
- Rebound jump height was the only predictor of reduced landing forces after adjusting for body weight and kit, highlighting its importance in safe landings.
- Extensor carpi radialis was the most active muscle during all descents, whereas biceps brachii activation increased with kit, reflecting altered muscular demands.

## 1 Introduction

Fast roping is a method of rapid entry into a field of operation used by elite law enforcement, military and special force operators. The field of operation could be descending through the roof of a building or a moving helicopter onto a ship, for example. The nature of this task is complex and physically demanding, but it is an important element in the role of these elite operators (Walker et al. 2017). Descents can range from 20 to 40 ft+ and can be significantly impacted by technique and equipment (Walker et al. 2017; Goldmann et al. 2014; Kragh and Taylor 1995; Glancy 1998). This exposes operators to descent times previously found between three and five seconds (Goldmann et al. 2014), with controlled descents suggested at a rate of 10–15 ft per second (Kragh and Taylor 1995). However, there is an absence of empirical data supporting the physical demands of fast roping, outside of the use of heart rate (HR) observations, which suggest that Royal Marine Commandos consistently attained > 80% maximal estimated HR in a simulated vessel boarding task (Walker et al. 2017).

Considering the complexity of the task, previous observations have stated an injury occurrence rate as low as 1%, with ankle and foot, spinal and knee injuries accounting for 45%, 12% and 11% of these, respectively (Kragh and Taylor 1995). However, the rate and severity of injury are significantly determined by the control of the descent, with uncontrolled descents consisting of speeds > 15 ft p/second. This was noted as due to loss of grip or missing the rope completely, showing a 100% rate of injury occurrence (Kragh and Taylor 1995), leading to guaranteed severe to catastrophic injuries (Kragh and Taylor 1995; Glancy 1998; Wolski et al. 2023). These included life‐altering injuries to the lumbopelvic region, lower limbs or ultimately death (Kragh and Taylor 1995; Glancy 1998). Due to the scarcity of research, it is intuitive to conclude from research looking at associated tasks such as parachuting, which see higher injury occurrence rates in comparison: 1.48 to 3.76 per 1000 parachute jumps (Zakowski et al. 2019) compared to 1 per 2000 fast rope descents (Kragh and Taylor 1995) respectively. Injury sites and severity are similar, with ankles and knees being the prominent site of injury (68%), typically resulting in temporary incapacity to work (Zakowski et al. 2019). It is worth noting that even though the occurrence of severe injuries is low, there was a 10% and 4% incident rate that removed personnel from service or caused permanent damage, respectively (Zakowski et al. 2019; Kragh and Taylor 1996). Causal factors that negatively influence the occurrence of injuries in parachutists include body weight (Bricknell and Craig 1999), weight of kit (Zakowski et al. 2019; Kragh and Taylor 1996; Bricknell and Craig 1999), improper landing position (Ellitsgaard 1987) and obstacles (Bricknell and Craig 1999; Ellitsgaard 1987) whereas experience (Zakowski et al. 2019; Kragh and Taylor 1996) and the individual operatives' strength (Zakowski et al. 2019) both show a positive influence in reducing injury risk.

A comprehensive understanding of upper limb muscular activity during fast roping descents is not yet known, nor are the corresponding landing forces after a successfully controlled descent. As previously mentioned, severe and fatal injuries occur because of loss of grip or missing the rope, combined with potential injury‐causal factors including individuals' strength, the weight of additional kit or obstacles on landing, and so it is imperative to gain an understanding of which physical factors impact LEO's ability to descend the rope safely. The primary aim of this study is to investigate whether the addition of operational equipment would increase the physical demand for fast roping. The secondary aim is to understand whether there are upper or lower limb strength qualities the LEO currently possess that result in a better‐controlled descent.

## 2 Methods

### 2.1 Experimental Approach to the Problem

**FIGURE 1:**
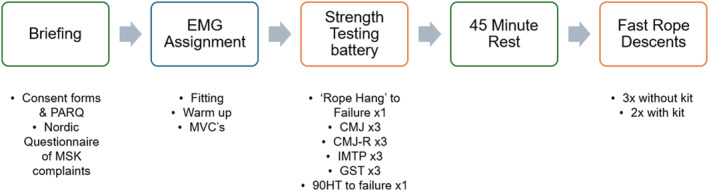
Data collection schematic.

This cross‐sectional study recruited operationally active LEOs as participants and contacted them via internal email through their national network and line managers. Participants (*n* = 37) were required for 1 day of testing (Figure 1) consisting of a briefing, screening, electromyograph (EMG) fitting (*n* = 12), a standardised instructor‐led warm‐up, maximal voluntary contractions (MVCs), strength testing and then five fast rope descents from a 20 ft platform. After an initial briefing, participants signed informed consent and were assigned a six‐digit alphanumeric code to maintain anonymity. Screening followed, with a PAR‐Q and ‘Nordic questionnaire of musculoskeletal complaints’ (Kuorinka et al. 1987) to establish EMG inclusion/exclusion according to the criteria in Table 1. Eligible participants were randomly assigned to either an EMG or no EMG group.

**TABLE 1:** Inclusion and exclusion criteria for assignment of EMG.

Inclusion criteria	No acute injuries (< 7 days since occurrence)
	Age between 18–55
	Operational law enforcement officer
	Successfully passed fast rope revalidation test
Exclusion criteria	Acute injury (e.g. Severe DOMS, recent sprain/strain)
	Serious underlying health issue (assessed via PAR‐Q)
	Failed fast rope revalidation test

### 2.2 Participants

A total of 37 LEO volunteered (*n* = 37; Age, 40 ± 5 years; Height, 180.61 ± 6.00 cm; Weight, 90.23 ± 10.64 kg); all demographic data is displayed in Table 2. Participants self‐reported their height and weight was collected at initiation of strength testing via Hawkin Dynamics Wireless Dual Force Platforms V3.0 (Maine, USA) at a sampling frequency of 1000hz (Badby et al. 2023; Dos’Santos et al. 2024). Ethical approval for the study was granted by The University College London Research Ethics Committee in line with the Declaration of Helsinki. This study was conducted in accordance with the EQUATOR Network Reporting Guidelines (STROBE) (Cuschieri 2019) (see checklist, Supporting Information S1: Digital content I).

**TABLE 2:** Participant demographics; including full sample (*n* = 37) and EMG sample (*n* = 12).

	Full sample Mean ± standard deviation	EMG sample Mean ± standard deviation
**Participants**	Male *n* = 36 Female *n* = 1	Male *n* = 12
**Ages (years)**	40 ± 5	41 ± 4
**Height (cm)**	180.6 ± 6.0	180.0 ± 5.5
**Bodyweight (kg)**	90.2 ± 10.6	91.1 ± 11.1
**Bodyweight + operational kit (kg)**	106.0 ± 11.1	106.8 ± 13.7
**Years as a law enforcement officer**	17 ± 4	18 ± 4
**Years fast roping**	5.1 ± 3.3	5.5 ± 4.4
**Enjoyment of fast roping (1–10)**	7.5 ± 2.2	
**Dominant hand.**		Left *n* = 0 Right *n* = 12

### 2.3 Procedures

All participants were fitted on their self‐reported dominant arm with Delsys Trigno Wireless EMG system (Massachusetts, USA). Youdas et al. (2010) and Rota et al. (2013), recommend the following muscles as the most reliable for EMG placement during upper‐body posterior chain exercises: flexor carpi radialis (FCR), extensor carpi radialis (ECR), biceps brachii (BB), pectoralis major (PEC) and latissimus dorsi (LAT). SENIAM guidelines (Hermens et al. 1999) were used to establish a standardised electrode placement, which consisted of shaving, abrasing and cleaning the skin with alcohol wipes, followed by securing the electrode to a site palpated on the muscle belly, with zinc oxide sports strapping. After electrode placement, participants completed the warm‐up followed by MVCs, which required maximal force/intent for 4 seconds against an immovable object as per previous research (Mananas et al. 2005; Hou et al. 2007; Lin et al. 2012; Serres and Enoka 1998; Gaudet et al. 2016; Castelein et al. 2015; Al‐Qaisi et al. 2022). This allowed EMG data normalisation and mitigated differences in freshness/fatigue between participants. EMG data was sampled at a rate of 2000hz, a bandpass filter of 40–400 was applied and then the signal was normalised with root‐mean‐square (RMS) to report as a %MVC. Muscle activation was then reported as total integrated EMG (iEMG), the area under the muscle activation curve during the entire duration of the task.

Strength testing (Figure 1) was initiated by completing a maximal effort ‘rope hang’ test time (HTT). This required LEO to isometrically hold their body two feet in the air on a 40 mm fast rope, with the grip maintained at sternum height without legs and feet contacting the rope. Participants then completed three repetitions of the countermovement jump (CMJ) (Souza et al. 2020), three repetitions of countermovement jump rebound (CMJ‐R) (Fahey et al. 2024; Xu et al. 2023), three six‐second maximal effort isometric midthigh pulls (Comfort et al. 2019) (IMTP), three maximal effort hand grip strength tests (GST) (Schaap et al. 2016) and one maximal effort 90‐degree hang test (90HT) (Clemons 2014) to failure. Each participant had one minute rest between repetitions (Clemons 2014) and five minutes rest between tests to negate the effects of fatigue (Parcell et al. 2002; Weir et al. 1994).

CMJ, CMJ‐R & IMTP were all conducted on Hawkin Dynamics Wireless Dual Force Platforms V3.0 (Maine, USA). Following (Heishman et al. 2020), participants were encouraged to use maximal force and intent. In agreement with Comfort et al. 2019 recommendations for IMTP, participants utilised a self‐selected posture replicating the start of the second pull of a clean. GST used a Jamar hydraulic hand dynamometer (Chicago, USA) and participants were instructed to follow the protocol established by Roberts et al. 2011. Finally, the 90HT was conducted on a pull‐up bar with participants adopting an isometric hanging position, elbows flexed at 90° and timed until failure. Failure was deemed as an observable increase in elbow flexion angle (> 90°) or volitional exhaustion. This has been found to be a reliable and valid measure of relative isometric strength and was theorised as a test that closely replicated the HTT (Clemons 2014; Clemons et al. 2004).

Participants completed five 20 ft descents on a 40 mm fast rope in a random order, three in standard uniform and two with the addition of an operational kit (kit weight: 15.8 ± 3.3 kg). The number of descents was limited by senior officers and fast rope instructors to not emit high levels of fatigue as LEOs were operationally active. For data analysis, unless otherwise stated, all values for the three descents in uniform were averaged per participant and are reported as descents ‘without kit’. All values for the two descents with full operational kit were averaged per participant and are reported as descents ‘with kit’. Participants' descents were validated against specific criteria (Supporting Information S1: Digital content II) and they had to land centrally on a 122 cm × 122 cm custom overlay on the Force Platforms. The ‘without kit’ descents had five minutes' rest between each and the ‘with kit’ conditions had 10 min. Force platform and EMG recording were initiated on the ‘GO’ command. Landing control (LC) was rated between one and five and recorded by instructors via a checklist (Supporting Information S1: Digital content III). Due to logistical constraints, it was not possible to conduct test‐retest repeatability for this checklist, however the same two instructors, both with 10+ years of fast rope instruction and 20+ years of operational experience, were used to provide evaluation for all participants on descents. Invalid descents were excluded due to incorrect technique or recording inaccuracy.

**FIGURE 2:**
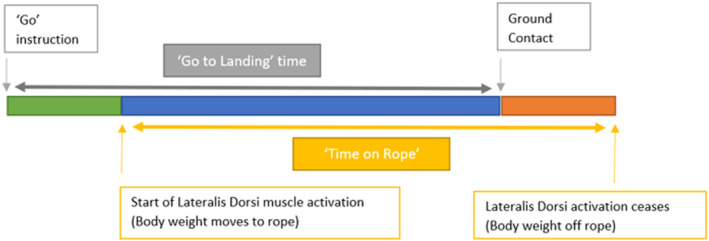
Time markers used to measure ‘Go to landing’ and ‘Time on Rope’ descent times.

Descent time was calculated twofold (Figure 2). ‘Go to landing’ time was from the moment the ‘GO’ command was given to the moment the participant contacted the force platform. This measurement, therefore, included ‘stalling’ before stepping off the platform. ‘Time on Rope’ descent time refers only to the time the participant spent on the rope itself. This was determined from the first significant increase in the EMG signal of the LAT muscle and concluded when the participant released the rope. LAT activation was used to determine ‘time on rope’ as this muscle gave the clearest increase in activation as the participants initiated their rope hold.

### 2.4 Statistical Analysis

Statistical analysis was carried out using Base‐R and RStudio (Lüdecke et al. 2021). *p*‐values < 0.05 were considered statistically significant. Normality was assessed using the Shapiro‐Wilk test. Outliers were excluded and residual normality was checked using histograms. A Paired *T*‐test or Wilcoxon Signed Rank test was used to determine the significant difference in variables between descent with and without kit. Independent *T*‐test or Mann‐Whitney *U* test was used to determine significant differences in means between categories. Stepwise multiple regressions were used to determine the predictive effect of anthropometric and strength variables on landing forces. Body weight was included as a covariate in models of landing forces because body mass directly influences impact magnitude; adjusting for weight allows estimation of other predictors' effects independent of size‐related variation.

## 3 Results

**FIGURE 3:**
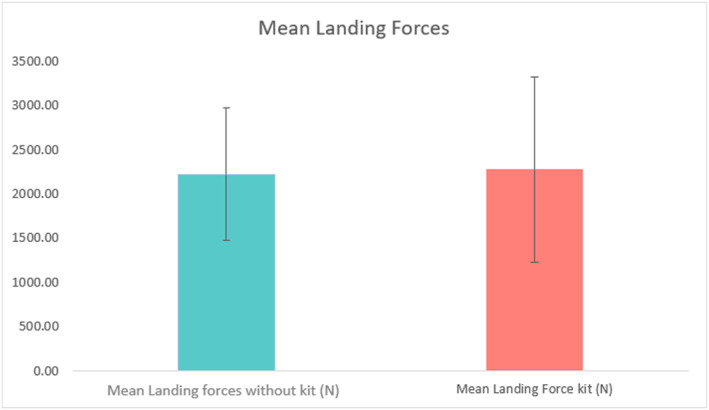
Mean landing forces for all participants without kit and with kit. No significant difference was observed between conditions.

There was no significant difference in mean landing forces between kit and without kit landings, 2270.66 ± 1048.17 N versus. 2218.39 ± 747.94 N, respectively (Figure 3).

**FIGURE 4:**
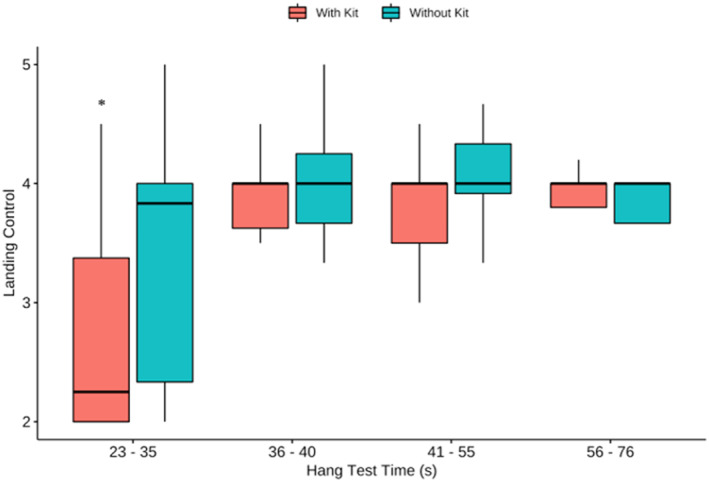
Mean landing control by hang test time (HTT). * Significant difference (*P* < 0.01) where participants with HTT < 35 s exhibited poorer landing control when descending with kit, compared to without kit. No difference between descents with or without kit.

Participants were grouped according to their HTT in order to evaluate the effects of HTT outcomes on LC. There were significant main effects for condition (F (2,32) = 7.5, *p* = 0.01) and HTT group (F (3,32) = 5.8, *p* = 0.003) and a condition*group interaction (F (3,32) = 3.33, *p* = 0.03) (Figure 4). Post hoc comparisons revealed that LEOs who achieved HTT of 23–35 s achieved poorer average LC scores compared to all other groups (*p* < 0.05). Specifically, they achieved lower scores when descending with kit compared to no kit (*p* < 0.001) and they achieved lower scores than all other LEOs particularly when descending with kit (*p* < 0.01). The group comparison did not reach significance when descending without kit. Therefore, LEOs who achieved a HTT inferior to 35 s performed significantly worse in their LC scores when wearing kit compared to all other groups.

**FIGURE 5:**
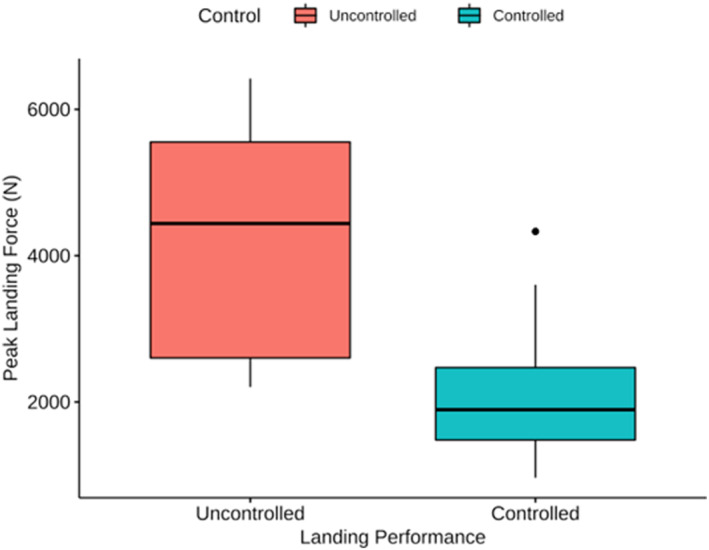
Mean ± SD peak landing force (includes all descents with and without kit), comparing uncontrolled and controlled landings. * Significant difference (*p* = 0.025).

Peak landing force was significantly higher (*p* = 0.025) in uncontrolled landings compared to controlled ones (Figure 5). There was no significant difference in peak landing force when descending with or without kit, nor was landing control affected by wearing kit; therefore, all descents with and without kit were included in this calculation, providing an average overall difference of 1681 N (Uncontrolled: 4244 ± 1827 N; Controlled: 2017 ± 728 N). Therefore, uncontrolled landings produced landing ground forces that were double those of controlled landings, regardless of kit.

**FIGURE 6:**
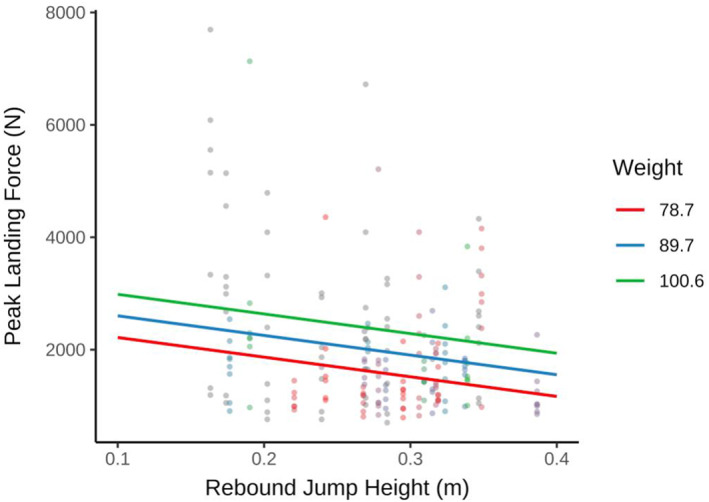
Predictive model output for the relationship between Rebound Jump Height (m), adjusted for weight and use of kit and Peak Landing Force (*N*).

To determine whether any lower limb strength and power measurements taken through the strength tests would influence descent landing forces, a stepwise regression was implemented using CMJ breaking impulse and rebound jump height as the main predictors in the unadjusted model (Model 1), subsequently adjusting for body weight and kit (Model 2, Table 3). While both higher CMJ breaking impulse and rebound jump height predicted lower landing forces in the unadjusted Model 1 (*R* ^*2*^ = 0.32, *p* = 0.009), only rebound jump height remained predictive with marginal significance (*p* = 0.06) after adjusting for body weight and use of kit (*R* ^*2*^ = 0.45, *p* = 0.008) (Figure 6).

**TABLE 3:** Stepwise regression models showing predictive variables that influence landing forces. Model 1 (*R* ^2^ = 0.32, *p* = 0.009) shows CMJ breaking impulse and rebound jump height as the main predictors in the unadjusted model. In Model 2 only rebound jump height remained predictive with marginal significance (*p* = 0.06) after adjusting for body weight and use of kit (*R* ^2^ = 0.45, *p* = 0.008).

Predictor	*b*	95% CI	*p*
Model 1			
(Intercept)	3280	[−1327, 7887]	0.15
CMJ breaking impulse	10	[0.45, 20]	0.04
Rebound jump height	−10232	[−20693, 229]	0.05
Model 2			
(Intercept)	−258	[−2657, 2148]	0.85
Weight	35	[13, 56]	0.007
Kit (Yes/No)	−10	[−71, 50]	0.75
CMJ breaking impulse	0.76	[−3, 4]	0.70
Rebound jump height	−3495	[−6813, −181]	0.06

*Note:* Model 1: Rsq = 0.32, *p* = 0.009. Model 2: Rsq = 0.45, *p* = 0.008.

**FIGURE 7:**
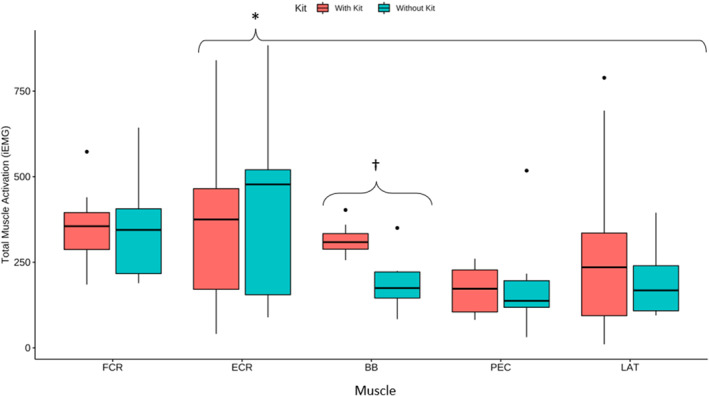
Total muscle activation (iEMG) for each muscle during drops without kit and with kit. ECR: Extensor Carpi Radialis, FCR: Flexor Carpi Radialis, BB: Biceps Brachii, PEC: Pectoralis Major, LAT: Latissimus Dorsi. † Significant difference for BB between kit and no kit (*p* < 0.05). * ECR is significantly more active than BB, PEC and LAT, regardless of kit (*p* < 0.05).

Due to friction with body armour and equipment, a significant amount of noise impacted the quality of EMG data for several participants. After discarding non‐usable data, a total final sample of 12 participants were included in the EMG analysis. There was a significant main effect of muscle (F (4,67) = 2.5, *p* = 0.05) where the ECR was significantly more active than the BB, PEC and LAT (*p* < 0.003). There was also a condition*muscle interaction (F (4,67) = 3.98, *p* = 0.006), where iEMG was significantly higher for the BB (*p* = 0.003) when descending with kit compared to without kit (Figure 7).

## 4 Discussion

Fast roping is a highly technical method of rapid entry into a scene of operation, with initial landing forces observed at ∼2200 N, whether with kit or without kit (Figure 3). It is evident that controlled landings do not exhibit high‐impact landing forces on elite LEOs. These data suggest that controlled landings had LEOs exposed to landing forces approximately 1.38–3.74x BW with kit and 1.66–3.35x BW without kit. With Mojaddarasil and Sadigh (2021) showing vertical ground reaction forces (vGRF) of up to 3.5x BW in bilateral jump landings, these results are not dissimilar to this study. Furthermore, contrary to our hypothesis, the addition of added kit weighing 15.8 ± 3.3 kg showed no significant increase in mean landing forces (Figure 3). Mojaddarasil and Sadigh (2021) stated that joint stiffness played a significant role in vGRF exposure and increased knee flexion angle resulted in a reduced vGRF to as little as 1.28x BW, but not necessarily reducing the internal load. This led to the inference that with low joint stiffness, the stress would shift away from the passive tissues, such as tendon and bone and result in a more controlled deceleration of the centre of mass through eccentrically loading the active musculature of the lower limbs (Mojaddarasil and Sadigh 2021). Interestingly CMJ Braking impulse, the rapid change in momentum applied to the body during the eccentric phase of initiating a jump, and rebound height, the ability to utilise the plyometric capabilities of the lower limb, where the only measures to correlate with landing forces before accounting for bodyweight and the addition of kit (Model 1, Table 3) (McMahon et al. 2018). This would suggest that the passive structures, which allow the neuromuscular system to take advantage of the stretch‐shortening cycle and turn deceleration into acceleration rapidly, are key physical measures for landing safely and dissipating the vGRF's upon impact. This may be particularly useful when considering the physical preparation for officers when training for fast‐rope descent without the addition of operational kit. However, when accounting for body weight and the addition of the operational kit (Model 2, Table 3), only rebound height shows marginal significance (*p* = 0.06), with confidence intervals all below zero. Rebound height seems to be a performance measure that, with improvement, can reduce the landing forces officers are exposed to. Interestingly, this model also shows body weight to be significant (*p* = 0.007), but with a small magnitude of effect, ∼35N reduction in landing force for each Kg of body weight. Model 2 seems to suggest that the addition of kit may change the kinematics of the landing mechanics without impacting the landing forces, which supports the idea that it is the active structures of the musculoskeletal system that dissipate the additional load of kit upon landing (Mojaddarasil and Sadigh 2021). This allows us to conclude that the landing forces for controlled descents are within a safe and tolerable range for LEOs when descending with and without kit. However, emphasis should be made to continue to land with low joint stiffness, to keep landing forces well within this range. LEOs should be trained and conditioned to tolerate increased eccentric braking forces and regularly use ballistic and plyometric exercises as part of their physical preparation to assist in the execution of fast‐rope descents safely.

Interestingly, having a longer hang test time (HTT) correlated with improved landing control (LC), particularly those LEOs that scored < 35's exhibited significantly poorer LC in kit (Figure 4). This suggests that training upper body isometric strength endurance could have a positive impact on LC. The HTT was analysed as an absolute measure rather than normalised to body weight because it represents an operationally relevant performance metric (e.g., a selection standard), increasing its ecological validity. This differs conceptually from adjusting for body weight in regression models, which were applied to account for its influence on landing forces rather than to redefine the performance metric. Notably, no physical strength or anthropometric variables alone correlated with or without kit descent mean landing forces. Given that the technical position for fast roping requires an isometrically contracted, fully flexed elbow, there is a risk that if the individual fails to maintain this position, the elbow may go through a heavy and rapid eccentric contraction. This could lead to a distal biceps tendon injury, which is uncommon but typically seen in active middle‐aged men following a heavy/rapid eccentric load from a semi‐flexed elbow position (Tarallo et al. 2018; Lacheta and Siebenlist 2018; Schmidt et al. 2013). Figure 7 shows a significant increase in iEMG for Biceps Brachii (BB) while wearing the kit. This may be due to the position of the hands being more distal from the chest, increasing the fulcrum at the elbow and stress on the BB, causing a greater need for supination to bring the rope back into the correct position at the sternum. Consideration should be taken in fast rope‐trained LEOs to strengthen their biceps through a full range of motion and to improve muscular endurance, focusing on muscular endurance through supination. Further to this, the Extensor carpi radialis (ECR) was significantly more active than all other muscles in all descents (Figure 7). With the ECR being of clinical significance in lateral epicondylitis injuries (Ahmad et al. 2013), it is worth noting that the high levels of ECR activation during regular training descents may increase injury risk within this population. However, within an analogous US military population, lateral epicondylitis has a low occurrence of 2.98 injuries per 1000 person‐years (Wolf et al. 2010). This being said, because ECR activity is so high, it is intuitive to include heavy gripping and carrying exercises, such as loaded farmers walk and isometric wrist extensions, into LEOs' training. These exercises have been shown to increase not only grip strength but also wrist extensor muscular endurance, improving tissue tolerance and reducing the risk of lateral epicondylitis (Shimose et al. 2011; Winwood et al. 2015).

Injury risk reduction requires understanding how the measured variables can be trained to help influence landing forces. A holistic approach to strength and conditioning, incorporating upper body training to improve the extensor carpi radialis and biceps brachii muscular endurance and lower limb training to improve braking impulse and rebound height, should improve the LEO's ability to tolerate the landing forces. Alongside this, monitoring of body composition to reduce any excess body fat and regular training exposure to this highly technical skill should increase LEO robustness to tolerate fast roping descents and increase landing control, which was a significant contributor to increasing peak landing forces.

When considering programming alongside traditional resistance training, the use of strongman‐based exercises such as ‘Farmers Walk’ or ‘Sled pull’ can make for efficient whole‐body strength sessions that positively impact grip muscular endurance (Keogh et al. 2014; Hindle et al. 2019). Additionally, the concurrent programming of ballistic exercises, for example, a countermovement jump focusing on a deeper and faster eccentric phase, achieving higher velocities of the individual's centre of mass before concentric acceleration, has been found to positively impact impulse (Sánchez‐Sixto et al. 2018; Nishiumi and Hirose 2024). This may not influence the rebound jump height (Nishiumi and Hirose 2024), therefore, the inclusion of plyometric exercises that improve ankle joint stiffness, which involve reducing ground contact time, has been shown to improve rebound jump performance (Zushi et al. 2022). The addition of five minutes of jump rope training 2–3x per week within LEO's warm‐up would be an efficient and effective strategy to include plyometrics that improves lower limb stiffness and, as a result, rebound height (García‐Pinillos et al. 2020).

Therefore, a whole‐body strength and conditioning program 2–3x per week, starting with five minutes of jump rope before completing ballistic exercises focusing on fast deceleration/braking forces and finishing with traditional resistance exercises or strongman training variations, ought to improve the physical qualities that impact landing forces for these tactical athletes.

There are a few limitations of this study to consider. The selection bias, the use of fast rope‐trained LEOs, was inevitable to avoid the risk of injury during descents. Another limitation was having a 122 cm × 122 cm landing area for LEOs to aim for, potentially slowing their descent. However, this also gives stronger ecological validity to the study, as operational descents might require targeting a particular landing zone. Going forward, this could be addressed with the introduction of force‐measuring insoles. It should also be noted that only one female participated in the study and a small sample of only 12 were eligible for EMG data capture; this may influence potential findings. The limited sample size was due to a number of factors, including a small initial pool of specialist LEOs who were trained for the task and limited time availability of those who volunteered to take part. With a growing number of female LEOs, future research should address potential gender differences in the physical requirements of the task and capture the biomechanical analysis of landing mechanics, with a particular focus on joint kinematics, to further establish safe landing strategies and guide strength and conditioning programs.

Fast Roping is a complex and technical task that, when completed with controlled descents, showed no significant difference between landing forces with or without the additional weight of the operational kit. Those participants with a hang test time greater than 35 s displayed increased levels of landing control. The extensor carpi radialis was the most active muscle of the upper limbs when descending. Further to this, the additional weight of the operational kit increases only the activity of the biceps brachii, altering the muscular demands of controlling a fast rope descent. CMJ breaking impulse and rebound jump height are two trainable variables that can impact landing forces upon a successful and safe descent. The holistic management of officers tasked with completing fast roping should incorporate a training approach that allows for strength and conditioning of the whole body and regular descent training to reduce skill fade and increase landing control.

## Author Contributions

**Joseph Warwick:** conceptualisation, methodology, data collection, formal analysis, writing – original draft preparation, review and editing. **Sarita Harris:** data collection and processing, writing – review before publication. **Hannah Ranger:** data collection and processing, writing – review before publication. **Paul Read:** data collection, writing – review before publication. **Flaminia Ronca:** funding acquisition, supervision, formal data analysis, writing – review and editing.

## Funding

This study was funded by a large British Law Enforcement Service – This was under a confidentiality agreement and they wish to remain unnamed in any publication.

## Ethics Statement

The University College London Research Ethics Committee granted ethical approval for the study. (Ref ‐ 13985/006).

## Consent

All participants signed informed consent and were assigned a six‐digit alphanumeric code to maintain anonymity.

## Conflicts of Interest

The authors declare no conflicts of interest.

## Supporting information


Supporting Information S1

Figure S1

Figure S2

## Data Availability

Data can be requested from ethics@ucl.ac.uk and quote Project ID: 13985/006 or through the corresponding author upon reasonable request and in line with the legally binding confidentiality agreement between University College London and the counter‐signed British Law Enforcement Service.
