# Supplementary figures and images for: Physical Characteristics of Fast Roping in British Elite Law Enforcement Officers
Source: Eur J Sport Sci. 2026 Feb 5;26(3):e70134. doi: 10.1002/ejsc.70134 (PMC12875842; doi:10.1002/ejsc.70134)

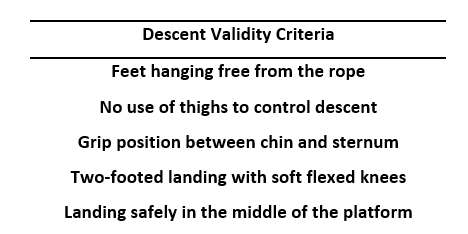

Supplement: Supplementary file 2 — Figure S1 [file EJSC-26-e70134-s003.png]

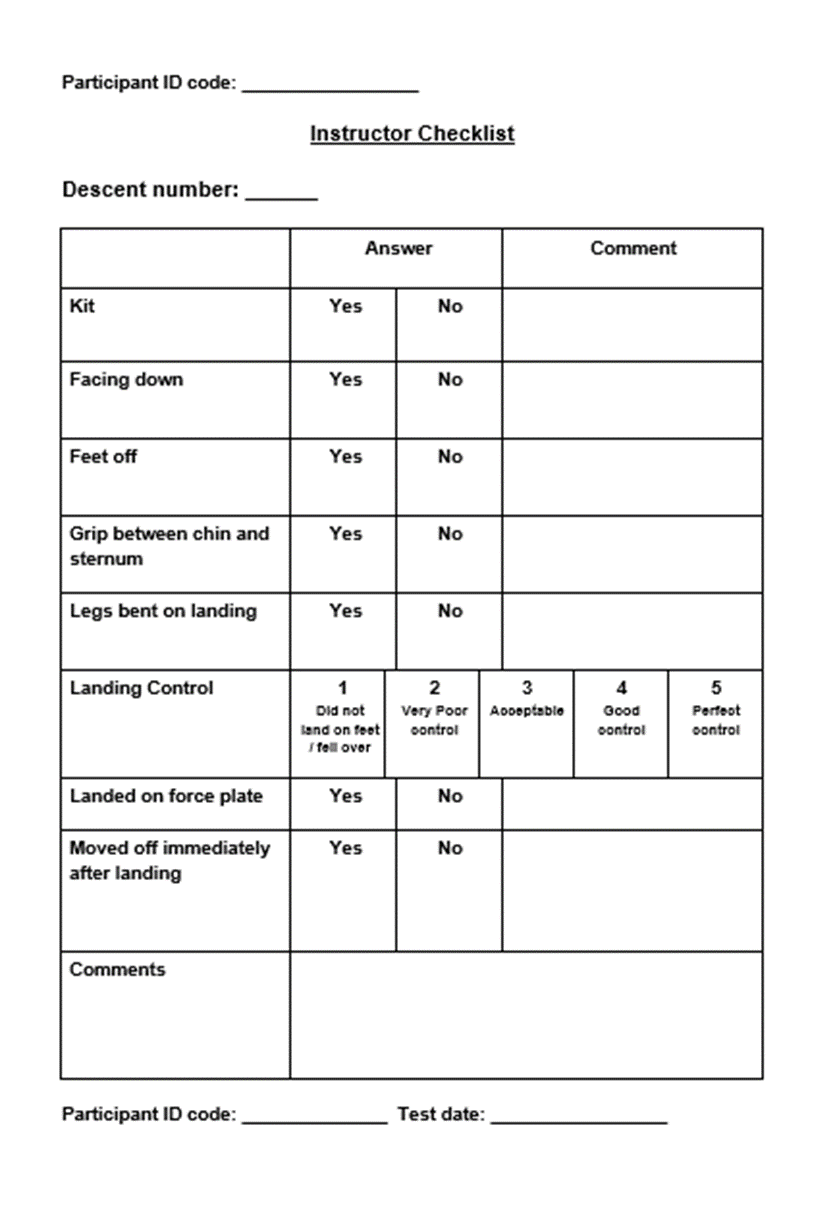

Supplement: Supplementary file 3 — Figure S2 [file EJSC-26-e70134-s001.png]
